# Exposure to vehicle traffic in childhood and lung function in young adulthood—a prospective cohort study in an area with low traffic-flows

**DOI:** 10.1186/s12940-025-01198-z

**Published:** 2025-07-07

**Authors:** Nicolás Bermúdez Barón, Helena Backman, Linnea Hedman, Eva Rönmark, Martin Andersson

**Affiliations:** Dept of Public Health and Clinical Medicine, The OLIN and Sunderby Research Unit, Umeå University, Umeå, Sweden

**Keywords:** Traffic-related air pollution, FEV_1_, FVC, Epidemiology, Spirometry

## Abstract

**Background:**

Exposure to high levels of vehicle traffic during childhood seems to have a negative effect on lung function. Less is known about the effects of exposure to relatively low levels during childhood. We aimed to study how exposure to vehicle traffic in childhood is associated with lung function and asthma in young adulthood in a 10-year follow-up of a population-based cohort in a municipality with relatively low levels of vehicle traffic.

**Methods:**

The Obstructive Lung Disease in Northern Sweden (OLIN) pediatric cohort II was recruited in 2006 at age 8 years. Exposure to vehicle traffic at baseline was studied in relation to lung function at follow-up at age 19 years (*n* = 1056 participants). Lung function measures included FEV_1_, FVC and FEV_1_/FVC. Different exposure thresholds were defined based on proximity (within a 200 m radius from the home address) to a road with a minimum daily count of heavy vehicles (≥ 250 and ≥ 500) or any type of vehicle (≥ 4000 and ≥ 8000). The association between exposure to vehicle traffic at baseline and lung function at follow-up was analyzed by linear regression adjusting for potential confounders.

**Results:**

In general, those above the exposure thresholds had lower lung function than those below, but not significantly so in all comparisons. Those exposed to ≥ 250 heavy vehicles/day had lower mean FEV_1_ z-score at follow-up (-0.38) compared with those exposed to < 250 heavy vehicles/day (-0.21), *p* = 0.033, and this association remained after adjustment for confounders (*p* = 0.036). Also, those exposed to ≥ 8000 vehicles/day had lower mean FVC z-score (-0.19) than those exposed to < 8000 vehicles/day (-0.02), *p* = 0.047, with *p* = 0.032 after adjustment.

**Conclusions:**

Exposure to vehicle traffic in childhood, in a relatively low traffic-flow environment, may be associated with a slightly lower lung function in young adulthood.

**Supplementary Information:**

The online version contains supplementary material available at 10.1186/s12940-025-01198-z.

## Background

Air pollution is one of the most important detriments of human respiratory health since the beginning of the Industrial revolution in the eighteenth century. Air pollution from land transports is known to have the highest impact on human respiratory health due to its proximity to urban centers [1, 2].

The World Health Organization (WHO) considers air pollution as the single largest environmental threat to human health and therefore has made efforts to persuade governments worldwide to improve the air quality by promoting policies that reduce human exposure to several key air pollutants such as particulate matter (PM) with an aerodynamic diameter of ≤ 2.5 μm (PM_2.5_), ≤ 10 μm (PM_10_), ozone (O_3_), nitrogen dioxide (NO_2_), sulfur dioxide (SO_2_) and carbon monoxide (CO) [3].

It is difficult to disentangle whether a specific pollutant is responsible for a specific effect, and they are all included in the term traffic-related air pollution (TRAP). There is evidence that as a mixture, air pollution may lead to oxidative stress and the generation of free radicals in the respiratory tissue causing airway inflammation, hyperresponsiveness, tissue damage and remodeling [4].

An individual’s lung function growth starts in utero and continues during childhood and young adulthood, reaching its peak around 20–25 years of age to subsequently decline by age. The development during childhood partially determines the lung function trajectory in early adulthood and later in life [5]. If lung function development during childhood or adolescence is negatively affected by factors such as TRAP exposure, it may lead to a below average or otherwise suboptimal future lung function trajectory [5–7], with increased morbidity and mortality risks [8–10].

Several European cross-sectional studies of air pollution exposure in early childhood have shown an association with lower lung function [11–13] with an even worsened outcome among those with allergic sensitization [14]. In addition, exposure to air pollution and heavy vehicle traffic has been associated with a higher risk of asthma, atopy and asthma symptoms such as wheeze during childhood [15–17], also in previous cross-sectional studies based on the cohort used in the current study [18].

There are also prospective multi-centre studies in regions with differing levels and type of air pollution showing the negative impact of TRAP exposure on lung function during childhood [11, 19] and others showing the beneficial effect of long-term reduction of air pollution exposure on the improvement on lung function in childhood and young adulthood [20, 21]. However, there are few studies from regions with relatively low levels of TRAP.

Our study aims to investigate the association between exposure to vehicle traffic-flow counts in childhood and lung function in young adulthood in a municipality of northern Sweden with relatively low traffic-flows.

## Methods

### The OLIN pediatric cohort II

The OLIN pediatric cohort II was established in 2006, when parents of all children in the 1st or 2nd grade of school (median age 8 years) in the municipalities of Luleå, Kiruna and Piteå (*n* = 2704) were invited to answer a questionnaire including the International Study of Allergy and Asthma in Childhood (ISAAC) questions [22] and additional questions about physician diagnosis of asthma, symptoms, asthma medications, and possible risk factors such as home dampness and parental smoking [23]. Among the respondents (n = 2585, 96% of invited) 1350 lived in Luleå, 726 in Piteå and 509 in Kiruna. In addition to the questionnaire, all respondents from Luleå and Kiruna did a skin prick testing (1700 participated, 90%, whereof 966 from Luleå). For the children living in Luleå, the geographic coordinates of the children’s home addresses were linked to vehicle traffic count data obtained from the local authorities [18].

**Fig. 1:**
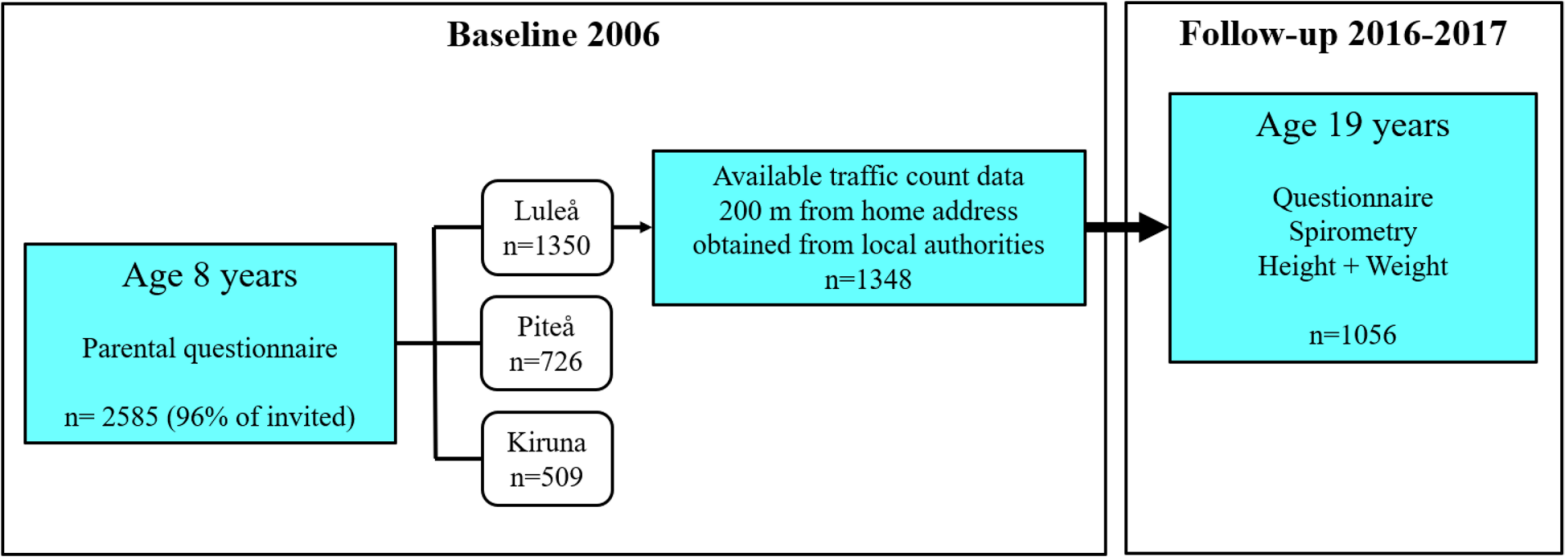
Study flow chart and participation in the OLIN pediatric cohort II

A follow-up at age 19 years was performed in 2016 and 2017 when participants were in the 12th year of school. This time they answered the questionnaire with additional questions about their own smoking habits. Those still living in Luleå and Kiruna were invited to a clinical examination that included spirometry, height and weight measurements. A total of 1470 (79% of the 1859 children who originally participated in the survey in 2006) participated in the spirometry in 2016–2017, of which 1056 were from Luleå constituting the study sample for our study (Fig. 1). Basic characteristics of those living in Luleå that participated at baseline but not at follow-up are presented in supplemental Table 1.

**Table 1:** Basic characteristics at baseline and follow-up

Baseline (age 8y)			Follow-up (age 19y)		
Mean Age (SD)		8.5 (0.5)	Mean Age (SD)		18.7 (0.4)
Sex (Girl)		543 (51.4)	Own smoking habits	Never smokers	888 (85.2)
Home dampness		121 (11.8)		Former smokers	48 (4.6)
Parental smoking		205 (19.8)		Current smokers	106 (10.2)
Physician-diagnosed asthma		55 (5.2)	Physician-diagnosed asthma		161 (15.2)
Current wheeze		90 (8.5)	Current wheeze		198 (18.8)
Any asthma medication last 12 months		72 (6.8)	Any asthma medication last 12 months		189 (17.9)
Allergic sensitization		293 (30.3)	BMI group	Underweight	69 (6.5)
Heavy vehicle traffic*	≥ 250	211 (20.0)		Normal weight	674 (63.8)
	≥ 500	117 (11.1)		Overweight	214 (20.3)
Total vehicle traffic**	≥ 4000	229 (21.7)		Obesity	99 (9.4)
	≥ 8000	154 (14.6)	Mean BMI (SD)		23.9 (4.7)

Expressed as n (%) unless otherwise stated. Study sample = 1056. SD: Standard deviation

^*^Heavy vehicle traffic in number of heavy vehicles/day. **Total vehicle traffic in number of vehicles/day

Allergic sensitization: Any skin prick test ≥ 3 mm. BMI: body mass index

Missing: Home dampness n = 28 (2.7). Parental smoking *n *= 23 (2.2). Allergic sensitization *n *= 90 (8.5). Own smoking habits *n *= 14(1.3)

### Vehicle traffic exposure at baseline

Vehicle traffic exposure was assessed based on the road with the highest mean number of passing vehicles within 200 m radius from the home address during 24 h on a weekday (not including Saturday or Sunday) when participants were 8 years old in 2006. Participants living in Luleå were exclusively studied as traffic-flow data was available only for this municipality. The participants’ home address was assigned to coordinates in a geographical information system (GIS) in which the traffic-flow data collected from 2006 ± 2 years from local traffic authorities was implemented. The data included counts of both heavy vehicles (trucks and buses that most often use diesel motors) and all vehicles (trucks, buses and cars) on all major roads and the majority of the smaller roads in the city center, while a large proportion of small roads within residential blocks were missing. The road with the highest vehicle count within 200 m radius was registered for each participant. Different traffic exposure thresholds were defined including heavy vehicle traffic (HVT) with ≥ 500 or ≥ 250 heavy vehicles/day and total vehicle traffic (TVT) with ≥ 4000 or ≥ 8000 vehicles/day [18].

### Lung function assessment at follow-up

At age 19 years, the participants performed a dynamic spirometry with a Jaeger Masterscope pneumotach spirometer following the 2005 ERS/ATS guidelines with a repeatability criterion of ≤ 150 ml [24].

This study used the spirometry-measured forced expiratory volume in 1 s (FEV_1_) and the forced vital capacity (FVC) without bronchodilatation, expressed as z-score (z) and as percent of predicted (pp) and also the FEV_1_/FVC ratio expressed as z-score (FEV_1_/FVCz) using the Global Lung Initiative reference values [25].

### Other factors

#### At baseline (age 8 years)

Home dampness was assessed by reporting dampness or mold inside the house either at the current or previous residence.

Parental smoking was based on whether the mother or the father or both reported smoking.

Physician-diagnosed asthma was defined by a positive answer to the question “Has your child been diagnosed by a physician as having asthma?”.

Current wheeze was defined by a positive answer to the question “Has your child had wheezing or whistling in the chest in the last 12 months?”.

Use of any asthma medication last 12 months was defined by answering “sometimes”, “often/periodically” or “every day” to the question “How often has your child had to use asthma medications in the last 12 months?”.

Allergic sensitization was defined as having any positive skin prick test (a mean wheal ≥ 3 mm) using a panel of 10 airborne allergens including birch, timothy, mugwort, dog, cat, horse, *Dermatophagoides farinae*, *Dermatophagoides pteronyssinus*, *Cladosporium* and *Alternaria* [23]. Of the study sample, 966 out of 1056 participated in skin prick testing.

#### At follow-up (age 19 years)

Physician-diagnosed asthma, current wheeze and use of asthma medication last 12 months were defined by the same questions as at baseline but this time addressed and answered by the participants. Own smoking habits were categorized as never smokers, former smokers and current smokers.

Height and weight were measured, and body mass index (BMI) was calculated by dividing the weight by the height squared (kg/m^2^) and was categorized as underweight (BMI < 18.5), normal weight (18.5 ≤ BMI < 25), overweight (25 ≥ BMI > 30) or obesity (BMI ≥ 30).

### Statistical analyses

The analyses were made with the IBM SPSS Statistics software, version 26 (IBM, Armonk, NY, USA). Comparisons of proportions across categorical variables were done by Chi-square tests. Comparisons of means of lung function variables between two groups were done by independent T-tests. *P*-values < 0.05 were considered statistically significant. Separate analyses were also done by stratifying the sample for allergic sensitization. The association between vehicle traffic exposure at baseline and lung function at follow-up was analyzed in separate linear regression models with each lung function variable (FEV_1_, FVC and FEV_1_/FVC) as outcome with adjustment for sex, home dampness, parental smoking and asthma diagnosis at baseline. Model covariates were selected based on previous knowledge, as illustrated by a directed acyclic graph in supplemental Fig. 1. The same regression models were also repeated excluding all participants with physician-diagnosed asthma at baseline, and by excluding all former and current (i.e. ever) smokers at follow-up.

## Results

The study sample included 1056 children of which 51.4% were girls. At baseline, mean age was 8.5 years, home dampness was reported by 11.8%, parental smoking by 19.8% and 30.3% had allergic sensitization confirmed by skin prick tests. In total, 5.2% of the participants had physician-diagnosed asthma, 8.5% reported current wheeze and 6.8% used any asthma medications last 12 months. Exposure to vehicle traffic at baseline showed that 211 (20.0%) participants lived close (within a 200 m radius) to a road with ≥ 250 heavy vehicles/day of which 117 lived close to a road with ≥ 500 heavy vehicles/day. Correspondingly, 229 participants (21.7%) lived close to a road with ≥ 4000 vehicles/day of which 154 with ≥ 8000 vehicles/day (Table 1).

At follow-up, 85.2% were never smokers, 10.2% current smokers and 4.6% former smokers. Physician-diagnosed asthma was reported by 15.2% of the participants, 18.8% reported current wheeze and any asthma medication was used by 17.9% in the last 12 months. The mean BMI of the study sample was 23.9 with most of the participants having a normal weight (63.8%), 99 participants (9.4%) were obese and 69 (6.5%) were underweight (Table 1).

Basic characteristics at baseline were additionally stratified by vehicle traffic exposure at baseline and showed that physician-diagnosed asthma, any asthma medication last 12 months and allergic sensitization were all significantly more common among those exposed to HVT ≥ 250 than in those exposed to HVT < 250. Further, home dampness, parental smoking and physician-diagnosed asthma were more common in those exposed to TVT ≥ 4000 than in those exposed to TVT < 4000 (Supplemental Table 2).

**Table 2:** Mean lung function at follow-up by characteristics at baseline

		FEV_1_z	FVCz	FEV_1_/FVCz
Sex	Boy	−0.09 (1.01)	0.06 (1.03)	−0.27 (0.99)
	Girl	−0.40 (0.96)	−0.14 (0.99)	−0.45 (0.97)
	*P*-value	** < 0.001**	**0.001**	**0.004**
Home dampness	No	−0.22 (1.00)	−0.02 (1.02)	−0.36 (0.98)
	Yes	−0.38 (0.98)	−0.20 (0.98)	−0.33 (0.98)
	*P*-value	0.106	0.068	0.738
Parental smoking	No	−0.21 (0.99)	−0.03 (0.99)	−0.33 (0.96)
	Yes	−0.37 (1.05)	−0.08 (1.11)	−0.48 (1.05)
	*P*-value	**0.036**	0.539	**0.045**
Physician-diagnosed asthma	No	−0.24 (0.99)	−0.05 (1.02)	−0.34 (0.97)
	Yes	−0.32 (1.09)	0.16 (0.86)	−0.73 (1.12)
	*P*-value	0.594	0.126	**0.004**
Current wheeze	No	−0.24 (0.97)	−0.05 (1.01)	−0.35 (0.96)
	Yes	−0.32 (1.20)	0.01 (1.09)	−0.51 (1.15)
	*P*-value	0.531	0.643	0.185
Any asthma medication last 12 months	No	−0.24 (0.99)	−0.05 (1.02)	−0.34 (0.97)
	Yes	−0.41 (1.03)	0.04 (0.96)	−0.69 (1.09)
	*P*-value	0.151	0.471	**0.004**
Allergic sensitization	No	−0.23 (0.99)	−0.01 (1.01)	−0.37 (0.96)
	Yes	−0.31 (1.02)	−0.09 (1.05)	−0.37 (1.04)
	*P*-value	0.244	0.318	0.922

Expressed as mean (SD) unless otherwise stated. SD: Standard deviation. Bold values indicate *p* < 0.05

Study sample = 1056. FEV_1_z: Forced expiratory volume in 1 s as % z-score

FVCz: Forced vital capacity as z-score. FEV_1_/FVCz: FEV_1_/FVC ratio z-score

Allergic sensitization: Any skin prick test ≥ 3 mm

Missing: Home dampness n = 28 (2.7). Parental smoking *n* = 23 (2.2). Allergic sensitization n = 90 (8.5)

Regarding mean lung function at follow-up, girls had significantly lower FEV_1_z, FVCz and FEV_1_/FVCz than boys, and those exposed to parental smoking at age 8y also had significantly lower FEV_1_z and FEV_1_/FVCz. FEV_1_/FVCz was also lower in participants with physician-diagnosed asthma and any asthma medication use last 12 months. In general, mean FEV_1_z was also lower, although with *p* > 0.05, for participants with reports of home dampness, physician-diagnosed asthma, current wheeze, any asthma medication last 12 months and allergic sensitization. The trend was less clear for FVCz (Table 2).

When additionally stratifying lung function for own smoking at follow-up, ever smokers had a mean FEV_1_z (SD) of −0.16 (0.97) and never smokers −0.26 (1.00) with *p* = 0.265. Mean FVCz (SD) was 0.16 (0.91) among ever smokers and −0.07 (1.03) among never smokers with *p* = 0.009. Mean FEV_1_/FVCz (SD) was −0.54 (0.97) among ever smokers and −0.32 (0.98) among never smokers with *p* = 0.013.

**Fig. 2:**
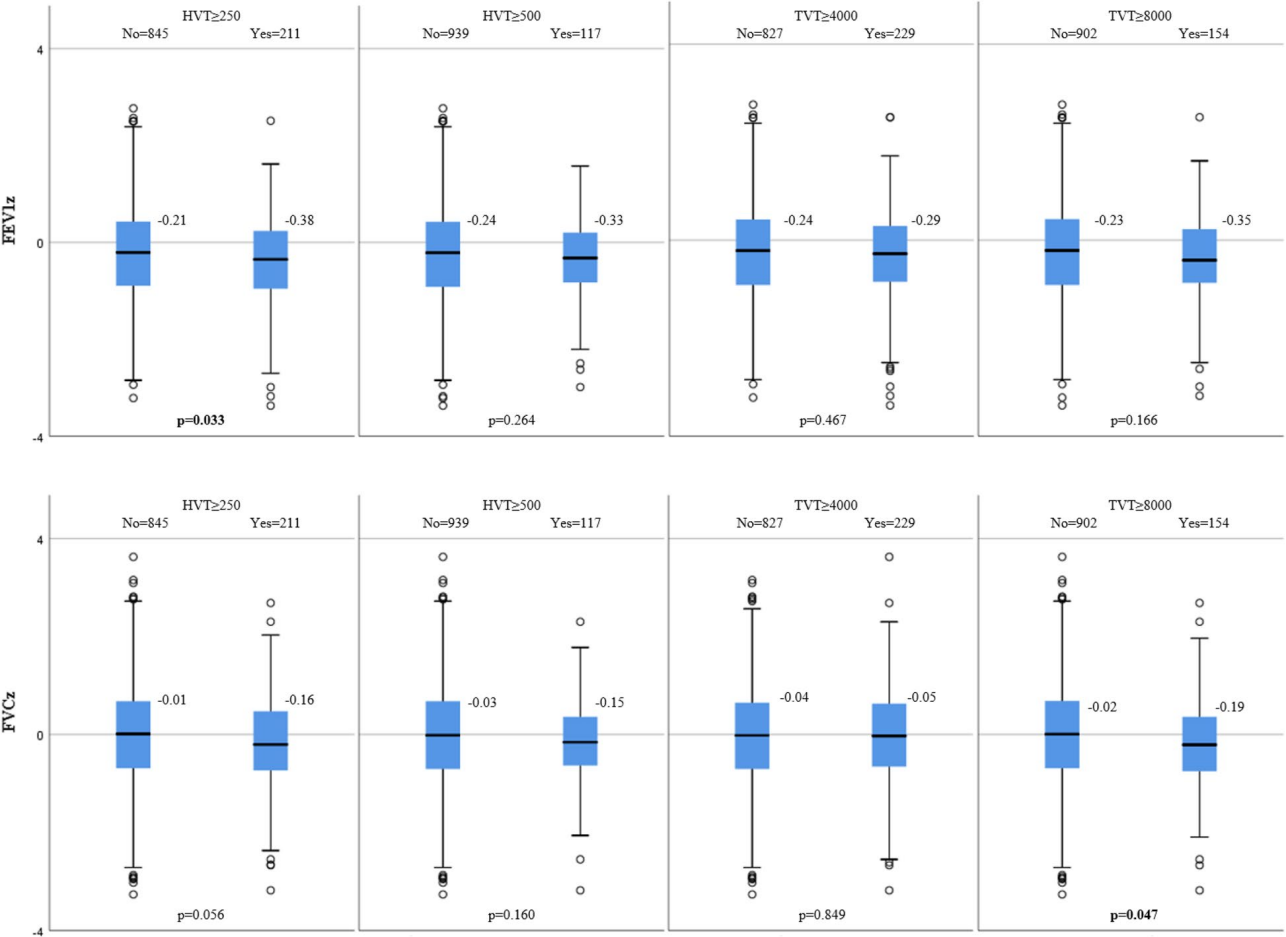
FEV_1_z and FVCz at follow-up by vehicle traffic exposure at baseline. *p* < 0.05 from T-tests in bold. FEV_1_z: Forced expiratory volume in 1 s as z-score. FVCz Forced vital capacity as z-score. HVT: Heavy vehicle traffic in number of heavy vehicles/day. TVT: Total vehicle traffic in number of vehicles/day

In general, although most often with *p* > 0.05, lower lung function values were seen in those with a vehicle traffic exposure above the defined thresholds compared to those below them. Regarding statistically significant findings, those exposed to HVT ≥ 250 had a lower mean FEV_1_z at follow-up (−0.38) in comparison to those exposed to HVT < 250 (FEV_1_z −0.21), and those with an exposure of TVT ≥ 8000 had a lower mean FVCz (−0.19) than those exposed to TVT < 8000 (FVCz −0.02) (Fig. 2). Similar results were seen for FEV_1_pp and FVCpp (Supplemental Fig. 2). No trends were seen for FEV_1_/FVCz.

In general, when stratifying the sample by allergic sensitization at baseline, those sensitized had lower FEV_1_z, FVCz, FEV_1_pp and FVCpp in comparison with the non-sensitized. The trend of lower lung function values in those above vehicle traffic exposure thresholds compared to those below was mainly seen in participants with allergic sensitization, although most comparisons did not reach statistical significance (Supplemental Table 3).

**Fig. 3:**
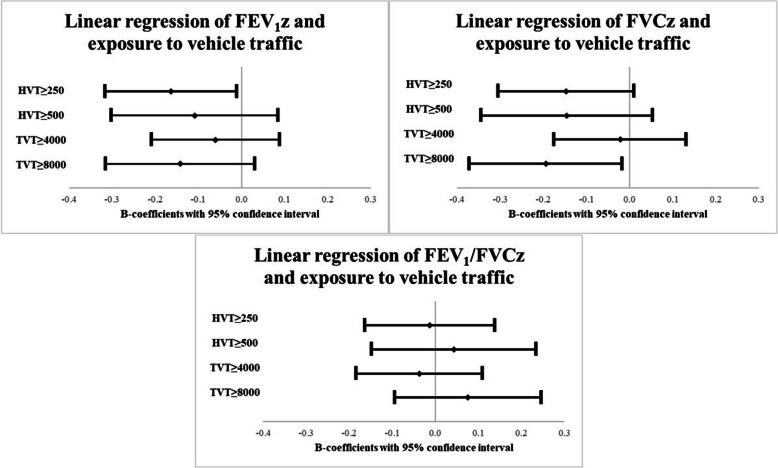
Adjusted associations between vehicle traffic exposure at baseline and lung function at follow-up. Adjusted B-coefficients from linear regression models are presented along with 95% confidence intervals. Each grade of exposure was included in separate models adjusted for sex, home dampness, parental smoking and physician-diagnosed asthma at baseline. FEV_1_z: Forced expiratory volume in 1 s as z-score. FVCz: Forced vital capacity as z-score. FEV_1_/FVCz: FEV_1_/FVC ratio z-score. HVT: Heavy vehicle traffic in number of heavy vehicles/day. TVT: Total vehicle traffic in number of vehicles/day

The lung function estimates presented in Fig. 3 were adjusted for sex, home dampness, parental smoking and physician-diagnosed asthma at baseline. Here the association between FEV_1_z and exposure to HVT ≥ 250 (*p* = 0.036), and between FVCz and exposure to TVT ≥ 8000 (*p* = 0.032) remained. In addition, the estimate for FVCz regarding exposure to HVT ≥ 250 yielded *p* = 0.067. Similar results were seen for FEV_1_pp and FVCpp and these are presented in supplemental Fig. 3. No associations with vehicle traffic exposure were seen for FEV_1_/FVCz.

**Fig. 4:**
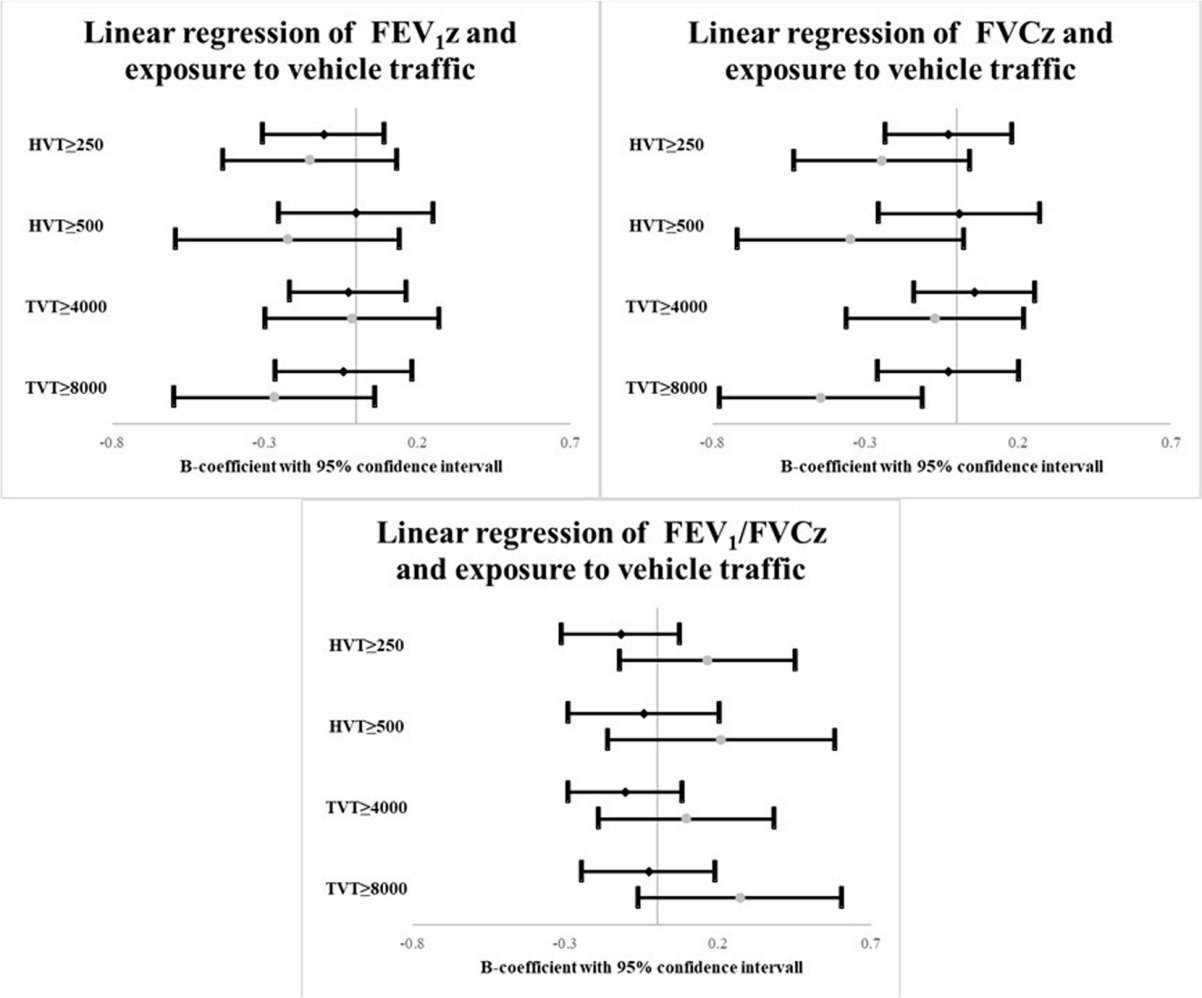
Adjusted association between vehicle traffic exposure and lung function stratified by allergic sensitization. Adjusted B-coefficients from linear regressions models are presented along with 95% confidence intervals. ♦ No allergic sensitization.  Allergic sensitization. Each grade of exposure was included in separate models adjusted for sex, home dampness, parental smoking and physician-diagnosed asthma at baseline. FEV_1_z: Forced expiratory volume in 1 s as z-score. FVCz: Forced vital capacity as z-score. FEV_1_/FVCz: FEV_1_/FVC ratio z-score). HVT: Heavy vehicle traffic in number of heavy vehicles/day. TVT: Total vehicle traffic in number of vehicles/day

When the linear regression models were stratified by allergic sensitization, those sensitized with an exposure of TVT ≥ 8000 had a significantly lower FVCz at follow-up of −0.44 units (*p* = 0.009) in comparison to those exposed to TVT < 8000. The same trends with lower FVCz were seen for participants with allergic sensitization exposed to HVT ≥ 250 and HVT ≥ 500, although with *p* > 0.05. No associations with vehicle traffic exposure were seen for FEV_1_/FVCz (Fig. 4).

**Fig. 5:**
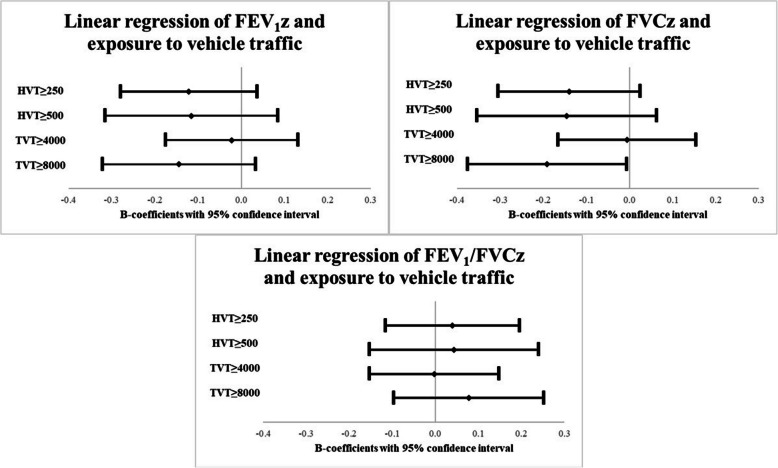
Adjusted associations between vehicle traffic exposure and lung function excluding participants with asthma. Adjusted B-coefficients from linear regressions models are presented along with 95% confidence intervals. Each grade of exposure was included in separate models adjusted for sex, home dampness and parental smoking at baseline. FEV_1_z: Forced expiratory volume in 1 s as z-score. FVCz: Forced vital capacity as z-score. FEV_1_/FVCz: FEV_1_/FVC ratio z-score). HVT: Heavy vehicle traffic in number of heavy vehicles/day. TVT: Total vehicle traffic in number of vehicles/day. Asthma = physician-diagnosed asthma at baseline (*n *= 55)

A sensitivity analysis was done by excluding all participants with physician-diagnosed asthma at baseline (*n* = 55) and is presented in Fig. 5 in which the trends were similar as in the total sample (Fig. 3). The association between exposure to HVT ≥ 250 and lower FEV_1_z lost significance, but the association between exposure to TVT ≥ 8000 and lower FVCz remained significant (B = −0.19 *p* = 0.043). No associations with vehicle traffic exposure were seen for FEV_1_/FVCz.

An additional analysis was done that excluded ever smokers at follow-up (*n *= 154) and is presented in supplemental Fig. 4. The association between exposure to HVT ≥ 250 and lower FEV_1_z remained significant (B = −0.19 p = 0.031), while the association between TVT ≥ 8000 and FVCz remained of similar magnitude but lost significance (B = −0.18 *p* = 0.071). FEV_1_/FVCz remained without significant associations.

## Discussion

This prospective study including more than 1000 children from the general population in a region with relatively low levels of vehicle traffic counts in 2006 at 8 years of age, revealed a general trend where children above exposure thresholds of ≥ 250 heavy vehicles/day, ≥ 500 heavy vehicles/day, ≥ 4000 vehicles/day and ≥ 8000 vehicles/day had a slightly lower lung function in terms of FEV_1_ and FVC when they were 19 years old. Additionally, these trends seemed to be stronger among children with allergic sensitization against airborne allergens. Of note, these trends only show slight differences in lung function and should be interpreted with caution.

Our study showed that exposure to vehicle traffic in childhood may be associated with a slightly lower lung function in terms of FEV_1_ and FVC but not FEV_1_/FVC in young adulthood. This early exposure may translate into adulthood, e.g. the maximum achievable lung function peak may be reduced or the risk for adult obstructive lung disease may increase. Previous reviews of several longitudinal studies have corroborated this where early life determinants including environmental exposures had a direct influence on an accelerated lung function decline. In other words, harmful early-life exposures impacts future adult respiratory health negatively and it is pivotal to reduce these [5–7].

Stratification for allergic sensitization implied that the observed association between traffic exposure and lower lung function mainly occur in sensitized individuals, although few of our tests reached statistical significance. Other studies have shown conflicting results. One study showed that children sensitized to common inhalant or food allergens had a more pronounced negative association between TRAP exposure and lung function [14]. A previous cross-sectional study of the current cohort found that vehicle traffic exposure increased the risk of wheeze, however, this was only found among non-sensitized children [18].

Air pollution is well-known to cause asthma and both asthma and air pollution associate with impaired lung function. In our study, those with asthma at baseline indeed had lower FEV_1_/FVC at follow-up than those without asthma. Thus, we adjusted for physician-diagnosed asthma at baseline and in addition, a sensitivity analysis was done in which all participants with physician-diagnosed asthma at baseline were excluded. In this case results remained similar as in the model without exclusion indicating that exposure to vehicle traffic is associated with lung function independently of asthma diagnosis at baseline.

Socioeconomic status is considered an important potential confounder as it may relate both to higher exposure to vehicle traffic due to limited access to a residence far from highly trafficked areas, and also have a negative impact on lung function as it associates with a wide variety of risk factors. We did not have data on socioeconomic status but could adjust for factors such as home dampness and parental smoking which can be considered as partial indicators of socioeconomic status. In our study, home dampness tended to be associated with lower lung function values, although none of the associations were statistically significant. Studies focused on the association between home dampness and lung function in children are scarce, but one US study on 12-year-old children showed a lower mid-expiratory flow rate of 1.0% among those exposed to home dampness [26]. Further, a previous study in the same area as the current study but on another cohort of 7-8y old schoolchildren showed that past or present home dampness associated with having asthma [27]. Regarding smoking habits, parental smoking at baseline was consistently and significantly associated with lower FEV_1_ at follow-up, regardless of traffic exposure, indicating that this is another factor that independently affects lung function. Own smoking at follow-up also seemed to contribute to lung function impairment even if participants only smoked for a relative short period of time due to their young age. In order to remove this effect on the association between vehicle traffic and lung function a sensitivity analysis was done by excluding ever smokers at follow-up, confirming the associations between heavy vehicle traffic and lower FEV_1_.

Our study has some limitations. Firstly, measurement of lung function was only done at follow-up when participants were 19 years old, not allowing for assessment of lung function development from childhood. It is also important to recognize that we used an indirect estimation of TRAP exposure through vehicle traffic counts which, unfortunately, was only assessed at baseline, limiting the assessment of the cumulative exposure during the study period as traffic counts may have varied through the years. However, as such changes probably are random, our data suggest that exposure at age 8y is of importance independently of exposure later in life. Personal exposure, although desirable, was not done due to limited resources. Additionally, the grade of exposure may be underestimated as some participants may have lived close to more than one road with high vehicle counts.

The use of vehicle traffic-flow counts as a proxy for TRAP exposure may also be a limitation. The Air Quality Data Management of the Swedish Meteorological and Hydrological Institute (SMHI) [28] published levels of NO_2_, SO_2_, PM_2.5_ and PM_10_, at baseline in 2006 but they were not solely traffic-related air pollutants. The method of indirectly measuring exposure to TRAP through vehicle traffic counts has however been widely used in multiple previous epidemiological studies where significant associations between residence distance from a pollutant source and health outcomes were found [29, 30]. The advantages of this method are that it is easier to implement, it requires less equipment, and it can be applied on different types of research settings.

The direct measurement of specific components of air pollution such as PM (≤ 2.5 or ≤ 10) or gases (CO_2_, NO_2_, etc.) by stationary sensors is also very common [11]. Usually, such sensors are placed in a school, a local police station, or other locations relatively close to the participant’s residences which implies that all participants close to the sensor are assumed to have the same grade of exposure. Here, modeling of exposure is an option, but was not within our resources for this study. Gauderman and colleagues did a study using this method and found that improvements in pollution levels of NO_2_, PM_2.5_, PM_10_ had significant positive effects on lung function growth from age 11 to 15 years old children, and both among children with and without asthma [21]. We could not discriminate between these pollutants in our study as we only had data on traffic-flow counts but still trends of increased traffic exposure and reduced lung function were found. Specifically regarding the region of our study, it is important to mention that concentrations of PM_10_ tend to be higher in northern Sweden where icy roads are common, thus requiring the use of studded tires at least half time of the year and traction sands on streets which cause an increase in road wear [31]. In 1999–2000 a study of a different cohort but in the same region as our study, outdoor air pollution was estimated both by traffic-flows and measured levels of outdoor NO_2_. The results showed that living close to high traffic-flows might increase asthma incidence but its association with NO_2_ was weak and nonsignificant. However, the effect of lung function was not evaluated in this study [32].

This study also has several strengths, including the longitudinal study design with high participation and retention rates. Further, stratification based on objective measurements of allergic sensitization was possible. The available data allowed for adjustment for smoking which is known to be associated with lung function outcomes. The population-based study design provides high external validity making results applicable and generalizable to other populations within relatively low traffic-flow regions. In addition, the prospective study design with a long follow-up time allows studying the associations between vehicle traffic exposure in childhood and lung function at age 19, i.e. close to the age when lung function peaks.

## Conclusions

In conclusion, this study indicated an association between childhood exposure to vehicle traffic and a slightly lower lung function in young adulthood and suggests the importance of implementing actions that improve air quality and reduce exposure to air pollution also when exposure is relatively low.

## Supplementary Information

Supplementary Material 1.

## Data Availability

The datasets used and/or analysed during the current study are available from the corresponding author on reasonable request.

## Abbreviations

WHO: World Health Organization
FEV_1_: Forced expiratory volume in 1 s
FVC: Forced vital capacity
FEV_1_/FVC: FEV_1_/FVC ratio
pp: % of predicted
z: z-score
PM_2.5_: Particulate matter with an aerodynamic diameter of ≤ 2.5 μm
PM_10_: Particulate matter with an aerodynamic diameter of ≤ 10 μm
O_3_: : Ozone
NO_2_: Nitrogen dioxide
SO_2_: Sulfur dioxide
CO: Carbon monoxide
CO_2_: Carbon dioxide
TRAP: Traffic-related air pollution
ISAAC: The International Study of Allergy and Asthma in Childhood
HVT: Heavy vehicle traffic
TVT: Total vehicle traffic
OLIN: The Obstructive Lung Disease in Northern Sweden studies.
ERS: European Respiratory Society
ATS: American Thoracic Society
BMI: Body mass index
SMHI: Swedish Meteorological and Hydrological Institute
