# Supplementary material for: Exposure to vehicle traffic in childhood and lung function in young adulthood—a prospective cohort study in an area with low traffic-flows
Source: Environ Health. 2025 Jul 7;24:44. doi: 10.1186/s12940-025-01198-z (PMC12235861; doi:10.1186/s12940-025-01198-z)
Supplement: Supplementary file 1 — Supplementary Material 1. [file 12940_2025_1198_MOESM1_ESM.docx]

**SUPPLEMENTAL MATERIAL**

**
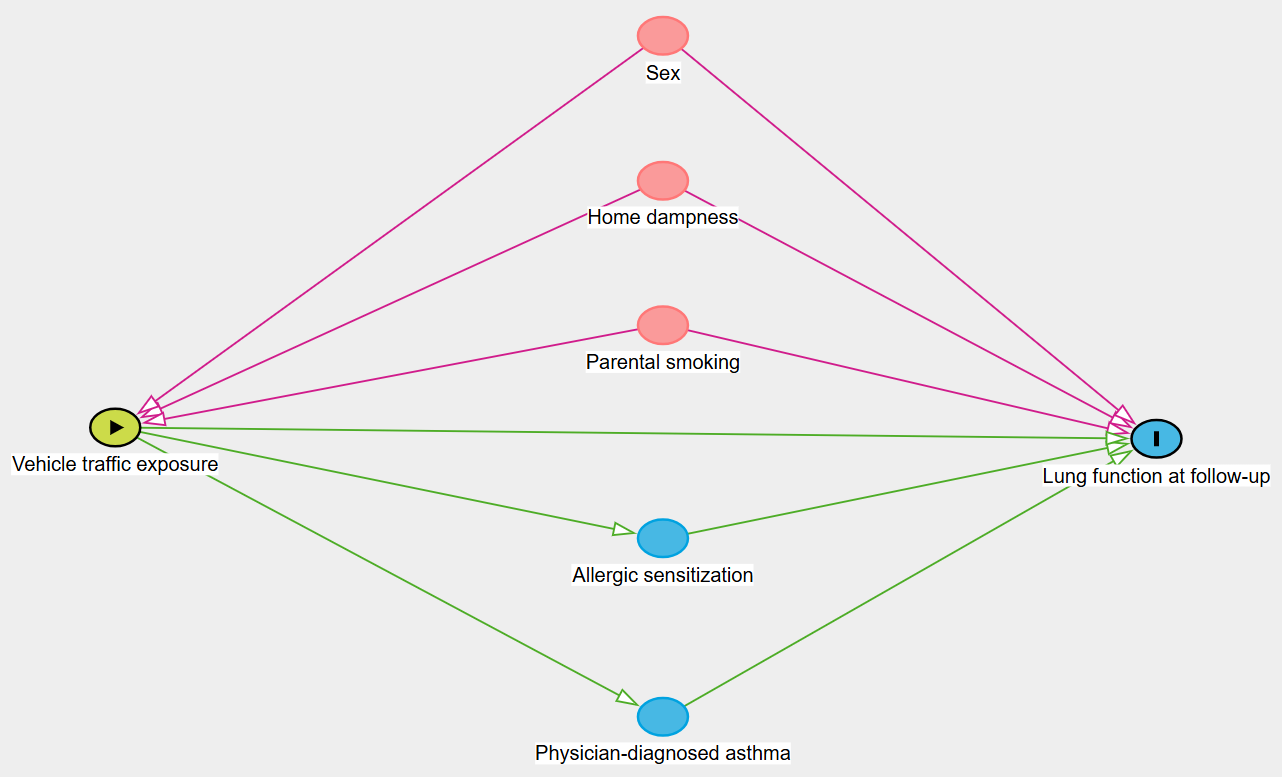
**

**Supplemental figure 1. Directed acyclic graph of potential confounders/mediators assessed at baseline related to the association between vehicle traffic exposure at baseline and lung function at follow-up.**


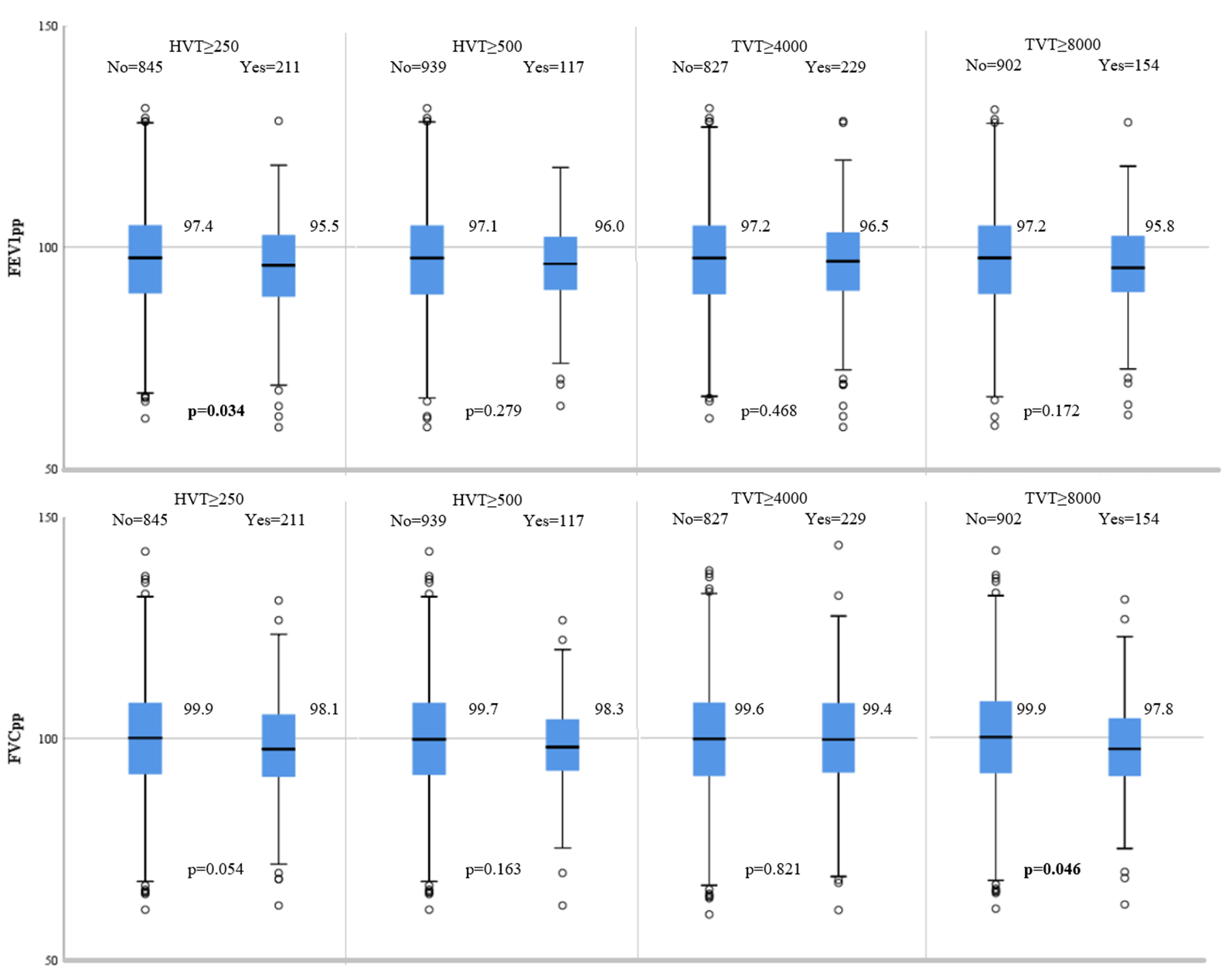


**Supplemental figure 2. FEV_1_pp and FVCpp at follow-up by vehicle traffic exposure at baseline.**

p<0.05 from T-tests in bold. FEV_1_pp: Forced expiratory volume in 1 second as % of predicted. FVCpp: Forced vital capacity as % of predicted. HVT: Heavy vehicle traffic in number of heavy vehicles/day. TVT: Total vehicle traffic in number of vehicles/day.


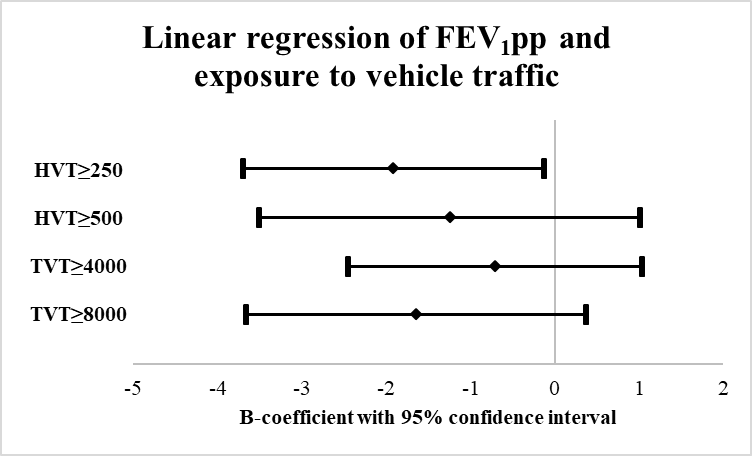

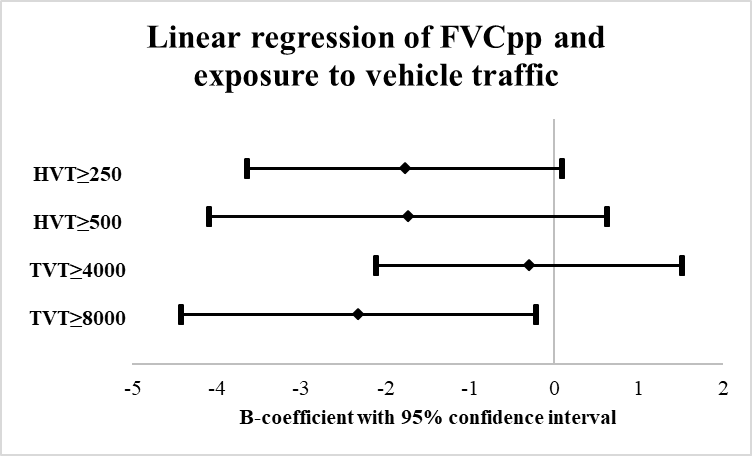


**Supplemental figure 3. Adjusted associations between vehicle traffic exposure at baseline and FEV_1_pp and FVCpp at follow-up.**

B-coefficients are presented along with 95% confidence intervals. Each grade of exposure was included in separate models adjusted for sex, home dampness, parental smoking and physician-diagnosed asthma at baseline. FEV_1_pp: Forced expiratory volume in 1 second as % of predicted. FVCpp: Forced vital capacity as % of predicted. HVT: Heavy vehicle traffic in number of heavy vehicles/day. TVT: Total vehicle traffic in number of vehicles/day.


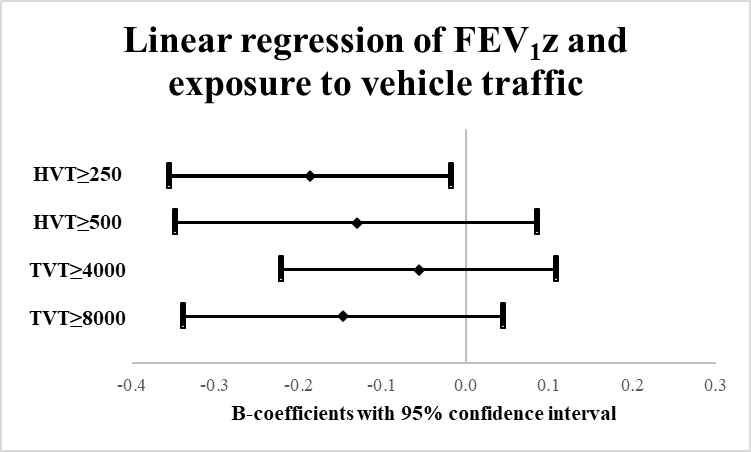

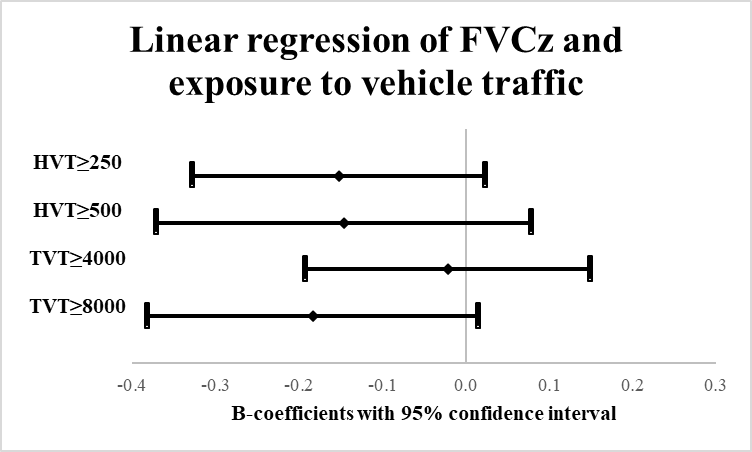

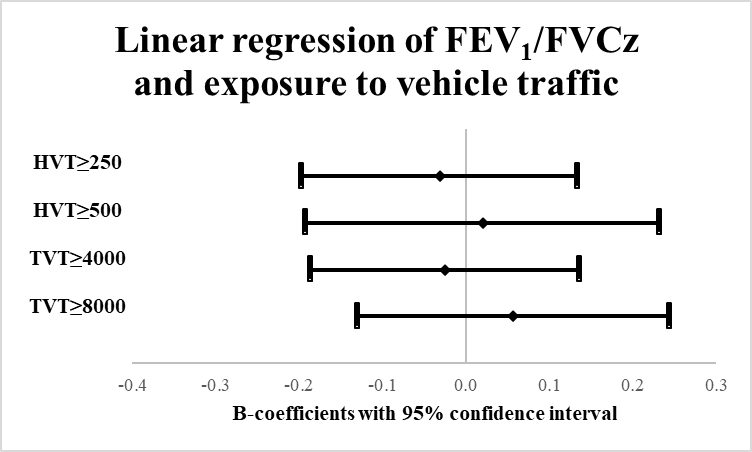


**Supplemental figure 4. Adjusted associations between vehicle traffic exposure and lung function excluding ever smokers at follow-up.**

Adjusted B-coefficients from linear regressions models are presented along with 95% confidence intervals. Each grade of exposure was included in separate models adjusted for sex, home dampness and parental smoking at baseline. FEV_1_z: Forced expiratory volume in 1 second as z-score. FVCz: Forced vital capacity as z-score. FEV_1_/FVCz: FEV_1_/FVC ratio z-score). HVT: Heavy vehicle traffic in number of heavy vehicles/day. TVT: Total vehicle traffic in number of vehicles/day. Ever smokers at follow-up = current smokers and former smokers at follow-up (n=154).

| **Supplemental table 1. Basic characteristics at baseline (age 8y) of non-participants at follow-up (age 19y)** | | |
| --- | --- | --- |
| Mean Age (SD) |  | 8.5 (0.6) |
| Sex (Girl) |  | 126 (43.2) |
| Home dampness |  | 41 (15.0) |
| Parental smoking |  | 81 (30.8) |
| Physician-diagnosed asthma |  | 23 (7.9) |
| Current wheeze |  | 37 (12.7) |
| Any asthma medication last 12 months |  | 25 (8,6) |
| Allergic sensitization |  | 70 (27.9) |
| Heavy vehicle traffic* | ≥250 | 71 (24.3) |
|  | ≥500 | 42 (14.4) |
| Total vehicle traffic** | ≥4000 | 82 (28.1) |
|  | ≥8000 | 51 (17.5) |
| Expressed as n (%) unless otherwise stated. Non-participants at follow-up=292. SD: Standard deviation. | | |
| *Heavy vehicle traffic in number of heavy vehicles/day.  **Total vehicle traffic in number of vehicles/day. | | |
| Allergic sensitization: Any skin prick test ≥3 mm. | | |
| Missing: Home dampness n=19 (6.5), Parental smoking n=29 (9.9), Allergic sensitization n=41 (14.0). | | |

| **Supplemental table 2. Basic characteristics at baseline by grade of traffic exposure at baseline (age 8y)** | | | | | | |
| --- | --- | --- | --- | --- | --- | --- |
|  | Heavy vehicle traffic≥250* | | | Heavy vehicle traffic≥500* | | |
|  | No=845 | Yes=211 |  | No=939 | Yes=117 |  |
|  | n (%) | n (%) | p-value | n (%) | Count | p-value |
| Home dampness | 92 (11.2) | 29 (14.3) | 0.214 | 102 (11.2) | 19 (16.7) | 0.085 |
| Parental smoking | 155 (18.7) | 50 (24.3) | 0.075 | 175 (19.1) | 30 (26.1) | 0.075 |
| Physician-diagnosed asthma | 37 (4.4) | 18 (8.5) | **0.015** | 46 (4.9) | 9 (7.7) | 0.200 |
| Current wheeze | 69 (8.2) | 21 (10.0) | 0.406 | 81 (8.6) | 9 (7.7) | 0.733 |
| Any asthma medication las 12 months | 51 (6.0) | 21 (10.0) | **0.043** | 61 (6.5) | 11 (9.4) | 0.240 |
| Allergic sensitization | 224 (28.9) | 69 (36.9) | **0.045** | 258 (29.9) | 35 (34.3) | 0.355 |
|  | Total vehicle traffic≥4000** | | | Total vehicle traffic≥8000** | | |
|  | No=827 | Yes=229 |  | No=902 | Yes=154 |  |
|  | n (%) | n (%) | p-value | n (%) | n (%) | p-value |
| Home dampness | 86 (10.6) | 35 (15.9) | **0.032** | 98 (11.1) | 23 (15.4) | 0.133 |
| Parental smoking | 148 (18.3) | 57 (25.3) | **0.020** | 172 (19.5) | 33 (21.9) | 0.503 |
| Physician-diagnosed asthma | 37 (4.5) | 18 (7.9) | **0.041** | 45 (5.9) | 10 (6.5) | 0.437 |
| Current wheeze | 69 (8.3) | 21 (9.2) | 0.692 | 79 (8.8) | 11 (7.1) | 0.507 |
| Any asthma medication las 12 months | 50 (6.0) | 22 (9.6) | 0.058 | 59 (6.5) | 13 (8.4) | 0.387 |
| Allergic sensitization | 222 (29.2) | 71 (34.3) | 0.161 | 248 (29.9) | 45 (33.1) | 0.451 |
| Expressed as n (%). Chi-square with p-values<0.05 in bold. Study sample=1056. | | | | | | |
| *HVT: Heavy vehicle traffic in number of heavy vehicles/day. **TVT: Total vehicle traffic in number of vehicles/day. | | | | | | |
| Allergic sensitization: Any skin prick test ≥3 mm. | | | | | | |
| Missing: Home dampness=28 (2.7). Parental smoking=23 (2.2). Allergic sensitization=90 (8.5). | | | | | | |

| **Supplemental table 3. Mean lung function at follow-up (age 19y) by vehicle traffic exposure at baseline (age 8y) stratified by allergic sensitization** | | | | | | | |
| --- | --- | --- | --- | --- | --- | --- | --- |
| No allergic sensitization |  | Heavy vehicle traffic≥250* | | | Heavy vehicle traffic≥500* | | |
|  |  | No=552 | Yes=121 |  | No=606 | Yes=67 |  |
|  |  | Mean SD | Mean SD | P-value | Mean (SD) | Mean SD | P-value |
|  | FEV_1_z | -0.22 (0.98) | -0.31 (1.00) | 0.283 | -0.22 (0.99) | -0.23 (0.96) | 0.942 |
|  | FVCz | -0.004 (1.03) | -0.06 (0.96) | 0.602 | -0.01 (1.03) | -0.02 (0.82) | 0.980 |
|  | FEV_1_/FVCz | -0.36 (0.95) | -0.44 (1.03) | 0.409 | -0.37 (0.96) | -0.41 (1.00) | 0.750 |
|  | FEV_1_pp | 97.5 (11.4) | 96.3 (11.7) | 0.283 | 97.3 (11.5) | 97.2 (11.2) | 0.951 |
|  | FVCpp | 100.0 (12.2) | 99.4 (11.4) | 0.599 | 99.9 (12.2) | 99.9 (9.9) | 0.997 |
|  |  | Total vehicle traffic≥4000** | | | Total vehicle traffic≥8000** | | |
|  |  | No=537 | Yes=136 |  | No=582 | Yes=91 |  |
|  |  | Mean SD | Mean SD | P-value | Mean SD | Mean SD | P-value |
|  | FEV_1_z | -0.22 (0.98) | -0.23 (1.03) | 0.920 | -0.22 (0.99) | -0.25 (0.99) | 0.786 |
|  | FVCz | -0.03 (1.01) | 0.03 (1.03) | 0.536 | -0.01 (1.03) | -0.05 (0.93) | 0.738 |
|  | FEV_1_/FVCz | -0.36 (0.95) | -0.44 (1.03) | 0.349 | -0.37 (0.96) | -0.37 (1.02) | 0.986 |
|  | FEV_1_pp | 97.3 (11.4) | 97.2 (11.9) | 0.911 | 97.3 (11.5) | 97.0 (11.6) | 0.790 |
|  | FVCpp | 99.7 (12.0) | 100.4 (12.2) | 0.551 | 99.9 (12.2) | 99.5 (11.0) | 0.737 |
| Allergic  sensitization |  | Heavy vehicle traffic≥250* | | | Heavy vehicle traffic≥500* | | |
|  |  | No=224 | Yes=69 |  | No=258 | Yes=35 |  |
|  |  | Mean SD | Mean SD | P-value | Mean SD | Mean SD | P-value |
|  | FEV_1_z | -0.27 (1.03) | -0.41 (0.99) | 0.319 | -0.28 (1.05) | -0.47 (0.79) | 0.206 |
|  | FVCz | -0.04 (1.04) | -0.24 (1.08) | 0.164 | -0.05 (1.06) | -0.35 (0.96) | 0.115 |
|  | FEV_1_/FVCz | -0.40 (1.04) | -0.27 (1.06) | 0.381 | -0.39 (1.05) | -0.20 (1.00) | 0.305 |
|  | FEV_1_pp | 96.7 (12.0) | 95.1 (11.6) | 0.328 | 96.6 (12.2) | 94.4 (9.4) | 0.219 |
|  | FVCpp | 99.6 (12.4) | 97.2 (12.8) | 0.163 | 99.5 (12.6) | 95.9 (11.4) | 0.111 |
|  |  | Total vehicle traffic≥4000** | | | Total vehicle traffic≥8000** | | |
|  |  | No=222 | Yes=71 |  | No=248 | Yes=45 |  |
|  |  | Mean SD | Mean SD | P-value | Mean SD | Mean SD | P-value |
|  | FEV_1_z | -0.30 (1.04) | -0.32 (0.97) | 0.874 | -0.27 (1.04) | -0.51 (0.88) | 0.155 |
|  | FVCz | -0.08 (1.06) | -0.12 (1.04) | 0.773 | -0.02 (1.05) | -0.43 (0.99) | **0.018** |
|  | FEV_1_/FVCz | -0.38 (1.05) | -0.33 (1.03) | 0.731 | -0.41 (1.05) | -0.14 (1.01) | 0.120 |
|  | FEV_1_pp | 96.4 (12.1) | 96.2 (11.4) | 0.889 | 96.7 (12.2) | 94.0 (10.5) | 0.159 |
|  | FVCpp | 99.2 (12.6) | 98.7 (12.2) | 0.764 | 99.8 (12.5) | 95.0 (11.7) | **0.018** |
| Expressed as mean (SD) unless otherwise stated. Bold values indicate p<0.05. Study sample=1056. | | | | | | | |
| *HVT: Heavy vehicle traffic in number of heavy vehicles/day. | | | | | | | |
| **TVT: Total vehicle traffic in number of vehicles/day. | | | | | | | |
| Allergic sensitization: Any skin prick test ≥3 mm. Missing Allergic sensitization=90 (8.5). | | | | | | | |
| FEV_1_z: Forced expiratory volume in 1 second as z-score. | | | | | | | |
| FVCz: Forced vital capacity as z-score. FEV_1_/FVCz: FEV_1_/FVC ratio z-score. | | | | | | | |
| FEV_1_pp: Forced expiratory volume in 1 second as % of predicted. FVCpp: Forced vital capacity as % of predicted. | | | | | | | |
